# Ligand-Specific Regulation of the Endogenous Mu-Opioid Receptor by Chronic Treatment with Mu-Opioid Peptide Agonists

**DOI:** 10.1155/2013/501086

**Published:** 2013-11-24

**Authors:** Marianna Murányi, Resat Cinar, Orsolya Kékesi, Erika Birkás, Gabriella Fábián, Beáta Bozó, András Zentai, Géza Tóth, Emese Gabriella Kicsi, Mónika Mácsai, Roberta Dochnal, Gyula Szabó, Mária Szücs

**Affiliations:** ^1^Institute of Biochemistry, Biological Research Center, Hungarian Academy of Sciences, P.O. Box 521, Szeged 6701, Hungary; ^2^Department of Pathophysiology, Faculty of Medicine, University of Szeged, Szeged, Hungary

## Abstract

Since the discovery of the endomorphins (EM), the postulated endogenous peptide agonists of the mu-opioid receptors, several analogues have been synthesized to improve their binding and pharmacological profiles. We have shown previously that a new analogue, *cis-1S,2R*-aminocyclohexanecarboxylic acid^2^-endomorphin-2 (ACHC-EM2), had elevated mu-receptor affinity, selectivity, and proteolytic stability over the parent compound. In the present work, we have studied its antinociceptive effects and receptor regulatory processes. ACHC-EM2 displayed a somewhat higher (60%) acute antinociceptive response than the parent peptide, EM2 (45%), which peaked at 10 min after *intracerebroventricular (icv)* administration in the rat tail-flick test. Analgesic tolerance developed to the antinociceptive effect of ACHC-EM2 upon its repeated *icv* injection that was complete by a 10-day treatment. This was accompanied by attenuated coupling of mu-sites to G-proteins in subcellular fractions of rat brain. Also, the density of mu-receptors was upregulated by about 40% in the light membrane fraction, with no detectable changes in surface binding. Distinct receptor regulatory processes were noted in subcellular fractions of rat brains made tolerant by the prototypic full mu-agonist peptide, DAMGO, and its chloromethyl ketone derivative, DAMCK. These results are discussed in light of the recently discovered phenomenon, that is, the “so-called biased agonism” or “functional selectivity”.

## 1. Introduction

Recently discovered endomorphin-1 (Tyr-Pro-Trp-Phe-NH_2,_ EM1) and endomorphin-2 (Tyr-Pro-Phe-Phe-NH_2_, EM2), which display high affinity and selectivity to mu-opioid receptors, proved to be effective against neuropathic and inflammatory pain with reduced side effects [1, 2]. Besides their essential pharmacophoric groups (Tyr^1^, Phe^3^, and the amidated C-terminus), they have a proline in the second position that serves as a stereochemical spacer. Substitution of Pro^2^ by alicyclic beta-amino acids has been shown to cause profound changes in bioactive conformation, proteolytic stability, and pharmacological activity [3]. Endomorphin-2 containing *cis*-(*1R,2S*)-ACPC^2^ residue (ACHC-EM2) displayed higher binding affinity, improved mu-receptor selectivity, and enhanced proteolytic stability compared to the parent peptide [3, 4]. These features make it a promising tool to study the conformational requirements of peptide binding to mu-receptor.

Opioids are among the most commonly used analgesics, but their clinical use is limited by the development of various unwanted side effects such as tolerance, physical dependence, and respiratory suppression. It has become apparent that analgesic tolerance and dependence are very complex phenomena, which involve changes at the cellular, neuronal, and system levels [5–9]. Traditionally, receptor desensitization (uncoupling the receptor and G-protein), internalization (trafficking the cell surface receptors into an intracellular compartment), and/or downregulation (decrease in the total number of receptors) have been proposed as possible mechanisms for tolerance [6–15]. Importantly, while endogenous opioid peptides efficiently desensitized and internalized mu-opioid receptors, the highly addictive morphine failed to induce measurable changes [11–18]. There are conflicting data whether there is a linear relationship between the intrinsic efficacy of a ligand and its ability to promote receptor internalization [19–22]. Whistler and colleagues have suggested that receptor endocytosis plays a protective role in reducing the development of tolerance [8, 15, 23]. Another new hypothesis is that opioid tolerance may also result from amplification or induction of an opioid receptor-coupled signal transduction pathway that is either poorly expressed or absent from opioid naive tissue [24]. Very recently, it has been revealed that the mu-opioid receptors, besides other G-protein coupled receptors, display “functional agonism,” also called “agonist-directed trafficking” or “biased agonism” implying that different agonists, while acting at the same receptor site, can induce distinct molecular changes and activate distinct downstream responses [25, 26].

Most works on opioid receptor trafficking were carried out in various *in vitro *cell models. The limitations of these models are obvious, including differences in cellular milieu and receptor expression levels. The study of endogenous opioid receptors using *in vivo *models has produced some interesting results that could not have been anticipated *in vitro *[27]. As part of a comprehensive work using various mu-opioid ligands of distinct efficacy, chemical nature, and abuse potential, we attempt to correlate receptor regulatory changes with analgesic tolerance. It has been shown that sustained morphine treatment of rats, leading to analgesic tolerance, did not change the density or G-protein coupling of the surface mu-opioid receptors. However, significant intracellular changes, including upregulation of the mu-sites and their cognate G-proteins, were noted in the light membrane fraction [28]. In addition, we have described that 14-methoxymetopon, an extremely potent, centrally acting mu-opioid specific analgesic with low tolerance, physical dependence, and other side effects, when given to rats either acutely or chronically, did not change the binding or G-protein signaling of mu-opioid receptors in rat brain subcellular membranes [29]. We have hypothesized that whereas surface opioid receptors and their cognate G-proteins mediate the acute effect of opioids, intracellular events may play a crucial role in the long-term changes elicited by chronic drug exposure [28].

In the present work, we have examined the new endomorphin analogue, ACHC-EM2, for its antinociceptive effect and receptor regulatory changes after acute and prolonged *in vivo* treatments. The model system used is based on subcellular fractionation of rat brains followed by ligand binding and functional measurements. Detailed characterization of the subcellular fractions by marker enzymes, electron microscopy, and receptor binding experiments has been reported [28–31]. To assess changes in the intracellular distribution of mu-opioid receptors, subcellular fractionation was combined with radioligand binding measurements. The degree of desensitization was estimated by ligand-stimulated guanosine-5′-O-(3-[*γ*[^35^S]hio)triphosphate, [^35^S]GTP*γ*S functional assays. It is concluded that the regulatory changes accompanying ACHC-EM2 analgesic tolerance are distinct from those of the prototypic mu-agonist peptide, DAMGO (Tyr-D-Ala-Gly-(NMe)Phe-Gly-ol), and its chloromethyl derivative, DAMCK (Tyr-D-Ala-Gly-(NMe)Phe-CH_2_Cl).

## 2. Materials and Methods

### 2.1. Chemicals

Endomorphin derivatives were synthesized as published [3]. DAMGO and [^3^H]DAMGO (36 Ci/mmol) were prepared and kindly provided to us by Drs. Farkas and Tóth (Biological Research Center, Szeged, Hungary) or purchased from Multiple Peptide System (San Diego, CA, USA). DAMCK was synthesized as published [32] by Anna Magyar (ELTE, Budapest, Hungary). [^35^S]GTP*γ*S was obtained from Isotope Institute Ltd. (Budapest, Hungary). Tris(hydroxymethyl)aminomethane, (Tris, free base), sodium chloride (NaCl), ethylenebis(oxyethylenenitrilo) tetraacetic acid (EGTA), guanosine 5′-diphosphate sodium salt (GDP), guanosine 5′-triphosphate sodium salt (GTP), guanosine 5′-[*γ*-thio]triphosphate tetralithium salt (GTP-*γ*-S-Li_4_), magnesium chloride hexahydrate (MgCl_2_ × 6 H_2_O), dithiothreitol, sucrose, and Nembutal were purchased from Sigma-Aldrich (St. Louis, MO, USA). Bradford reagent was from Bio-Rad Laboratories (Hercules, CA, USA).

### 2.2. Animals

Wistar rats (250–500 g) were used. They were kept under a standard light-dark cycle with food and water available *ad libitum*. The animals were kept and treated according to the rules of the Ethical Committee for the Protection of Animals in Research (University of Szeged and Biological Research Center of the Hungarian Academy of Sciences, Szeged, Hungary). All efforts were made to minimize their suffering during treatments. The animals were randomly assigned to treatment groups (*n* = 4/dose/treatment).

### 2.3. Surgery

For *intracerebroventricular (icv)* peptide administration, the rats were implanted with a stainless steel Luer cannula (10 mm long) aimed at the right lateral cerebral ventricle under Nembutal (35 mg/kg, *intraperitoneally*) anesthesia [32]. The stereotaxic coordinates were 0.2 mm posterior, 1.7 mm lateral to the bregma, and 3.7 mm deep from the dural surface, according to the atlas of Pellegrino et al. [33]. Cannulas were secured to the skull with dental cement and acrylate. The experiments were started 5 days after *icv* cannulation. All *icv* treatments were delivered to freely moving animals to prevent unspecific effects. Upon conclusion of the experiments, 10 *μ*L of methylene blue was injected into the ventricle of decapitated animals and the position of the cannula was inspected visually. Animals with improper cannula placement were excluded from the final statistical analysis.

### 2.4. *In Vivo* Opioid Treatments

The peptides were dissolved in artificial cerebrospinal fluid (CSF) and injected in a volume of 2 *μ*L. Endomorphins were administered either acutely at the indicated doses (100 pg–20 *μ*g) or chronically at 20 *μ*g for 10 days (twice daily) and tested at the indicated times. Vehicle control groups obtaining CSF were used in all experiments, and no changes were detected in the antinociceptive response of the control group. DAMGO and DAMCK were injected at 10 *μ*g twice daily for 8 days. Animals were killed 16 hrs after drug treatments by decapitation immediately followed by subcellular fractionation.

### 2.5. Tail-Flick (TF) Antinociceptive Assay

The original method of D'Amour was used to determine analgesia in the rat by measuring the time required to respond to a radiating heat stimulus [32]. A beam light was focused on the tip of the tail, and the latency required for the rat to remove its tail was determined before (baseline) and after drug administration. The animals were tested at the times shown after injection of the peptides. The antinociceptive effect was expressed according to the equation: (TF_*n*_ − TF_*o*_) × 100/TF_max⁡_ − TF_*o*_, where TF_*o*_ is the tail-flick latency before drug administration, TF_*n*_ is the value of a repeated corresponding measurement after drug injection, and TF_max⁡_ indicates the cutoff time which was set at 10 sec in our assays. Statistical analysis of the data of tail-flick antinociceptive test was made by ANOVA. For significant ANOVA values, groups were compared by Tukey's test for multiple comparisons with unequal cell size. A probability level, *P* < 0.05, was accepted to label significant differences.

### 2.6. Crude Rat Brain Membranes

Crude brain membranes were prepared as published [34]. Briefly, pooled brain tissues without cerebella of four rats were washed with ice-cold buffer and their weight was measured. They were homogenized in 30 volumes (v/w) of ice-cold 50 mM Tris-HCl buffer (pH 7.4). Homogenates were centrifuged at 20,000 ×g for 25 min, and the resulting pellets suspended in buffer and spun again. Pellets were taken up in the original volume of buffer and incubated for 30 min at 37°C, followed by centrifugation at 20,000 ×g for 25 min. The supernatants were carefully discarded, and the final pellets were taken up in 5 volumes (v/w) of 50 mM Tris-HCl buffer (pH 7.4) containing 0.32 M sucrose. Appropriate membrane aliquots were stored at −80°C for several weeks. Prior to the binding experiments, an appropriate aliquot was thawed, diluted with 5-fold Tris-HCl buffer, and centrifuged at 20,000 ×g for 25 min to remove sucrose. The resulting pellets were taken up in 50 mM Tris-HCl buffer (pH 7.4) to yield in 0.3–0.5 mg membrane protein/mL.

### 2.7. Subcellular Fractionation of Rat Brains

Subcellular fractions of rat brains were purified as published [28, 29]. Briefly, fresh forebrains of four rats were pooled, gently homogenized in 10 volumes (v/w) of ice-cold buffer (5 mM Tris-HCl pH 7.4, 50 *μ*M CaCl_2_, 0.5 mM dithiothreitol), and supplemented with 10% sucrose. All sucrose solutions were made in the above buffer. The homogenate was spun at 1,000 ×g for 10 min. The resulting pellet was resuspended in the above buffer and spun again. The combined supernatants were spun at 12,000 ×g for 20 min. The pellets were suspended in 10% sucrose and subjected to consecutive centrifugations at 20,000 ×g for 25 min and 14,000 ×g for 20 min twice resulting in crude synaptic plasma membranes (SPM). The resulting pellets were lysed followed by fractionation on a 10%, 28.5%, and 34% sucrose density step gradient that was spun at 100,000 ×g for 2 h. Highly enriched SPMs were obtained from the 28/34% interface. Crude microsomal (MI) fractions were obtained from the 12,000 ×g supernatant by consecutive centrifugations at 20,000 ×g for 25 min and 100,000 ×g for 1 h. MI fractions were purified on a 10% and 28.5% sucrose step gradient centrifuged at 100,000 ×g for 2 h and collected at 10/28.5% interface. SPM and MI fractions were diluted threefold with 50 mM Tris-HCl buffer and pelleted at 100,000 ×g for 1 h to remove sucrose. The resulting pellets were taken up in 50 mM Tris-HCl (pH 7.4) buffer to yield in 0.3–0.8 mg membrane protein/ml and were freshly used.

### 2.8. Protein Concentration

The protein content of the membrane preparations was determined by the method of Bradford using bovine serum albumin as a standard [35].

### 2.9. [^3^H]DAMGO Binding Assay

Briefly, homologous displacement assays were performed by incubating [^3^H]DAMGO (*≈*1 nM) with 11 concentrations of unlabeled DAMGO (10^−10^–10^−5 ^M) and the membrane suspension (200–300 *μ*g protein) in 50 mM Tris-HCl (pH 7.4) buffer in a final volume of 1 mL as described [28, 29]. The tubes were incubated at 25°C for 1 h. The reaction was stopped by vacuum filtration through Whatman GF/C glass fiber filters (Whatman, Maidstone, England) using a Brandel M24-R Cell Harvester (Brandel, Gaithersburg, MD, USA). Filters were rapidly washed with 3 × 5 mL ice-cold 50 mM Tris-HCl (pH 7.4) buffer, air-dried, and counted in a toluene-based scintillation cocktail in a Wallac 1409 scintillation counter (Wallac, Turku, Finland). All experiments were performed in duplicate and repeated at least three times. Curves were constructed and analyzed by means of the GraphPad Prism 4 program (GraphPad Software, Inc., San Diego, CA, USA.) to obtain *K*_*D*_ (dissociation constant) and *B*_max⁡_ (receptor density) values. The data reported are means ± S.E.M. 

### 2.10. Ligand-Stimulated [^35^S]GTP*γ*S Functional Assay

The assay was performed as published [28, 29] with slight modifications. Briefly, rat brain membrane fractions (*≈*10 *μ*g of protein) were incubated with [^35^S]GTP*γ*S (0.05 nM) and 5-6 concentrations (10^−9^−10^−4 ^M) of opioid peptides in the presence of 100 *μ*M GDP in Tris-EGTA (50 mM Tris-HCl, 1 mM EGTA and 5 mM MgCl_2_; pH 7.4) buffer in a total volume of 1 mL for 60 min at 30°C. Nonspecific binding was determined with 10 *μ*M GTP*γ*S and subtracted. Bound and free [^35^S]GTP*γ*S were separated by vacuum filtration through Whatman GF/F filters using a Brandel M24-R Cell Harvester (Brandel, Gaithersburg, MD, USA). Filters were rapidly washed with 3 × 5 mL ice-cold 50 mM Tris-HCl (pH 7.4) buffer, air-dried, and counted in a toluene-based scintillation cocktail in a Wallac 1409 scintillation counter (Wallac, Turku, Finland). All assays were performed in triplicate and repeated at least three times. Curves were constructed and analyzed by fitting sigmoidal dose-response curves using the GraphPad Prism 4 program (GraphPad Prism Software Inc) to obtain potency (EC_50_, concentration of the ligand to give half-maximal effect) and efficacy (*E*_max⁡_, % maximal stimulation over basal activity) values. Basal activities were measured in the absence of opioid ligands and set as 0%. The data reported are means ± S.E.M.

## 3. Results

### 3.1. Antinociceptive Effect of ACHC-EM2 after Acute Treatments

**Figure 1:**
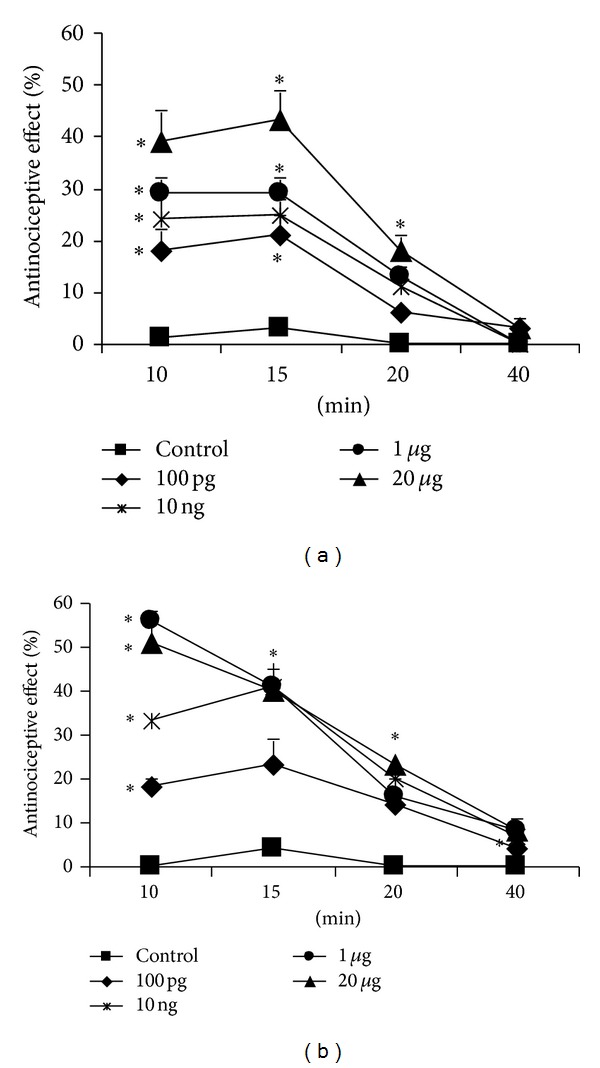
Time-course of the acute antinociceptive effect of graded doses of EM2 (a) and ACHC-EM2 (b) in rat tail-flick assay. The peptides were administered *icv* and assayed as described in Methods. Control groups received CSF. Data are shown as mean ± S.E.M; *n* ≥ 6. Statistical significance was determined by ANOVA and set at *P < 0.05 compared to appropriate control values.

In the first part of the study, the antinociceptive effect of the new ligand, ACHC-EM2, was evaluated and compared to that of its parent peptide, EM2, in tail-flick tests. Rats were injected *icv *with different doses of the peptides between 100 pg and 20 *μ*g as indicated in Figure 1. Animals treated with lower doses of ACHC-EM2 displayed a concentration-dependent increase (20–50%) in analgesic response compared to base-line values. The acute antinociceptive effect peaked around 10–15 min, followed by a rapid decrease, which dissipated by 40 min following injection (Figure 1). Higher doses (1 and 20 *μ*g) of ACHC-EM2 displayed around 60% antinociceptive responses which were somewhat higher than those of the parent peptide, EM2 (45%), which peaked at an earlier time (10 min).

### 3.2. Analgesic Tolerance Develops to the Antinociceptive Effect of ACHC-EM2 after Chronic Treatments

**Figure 2:**
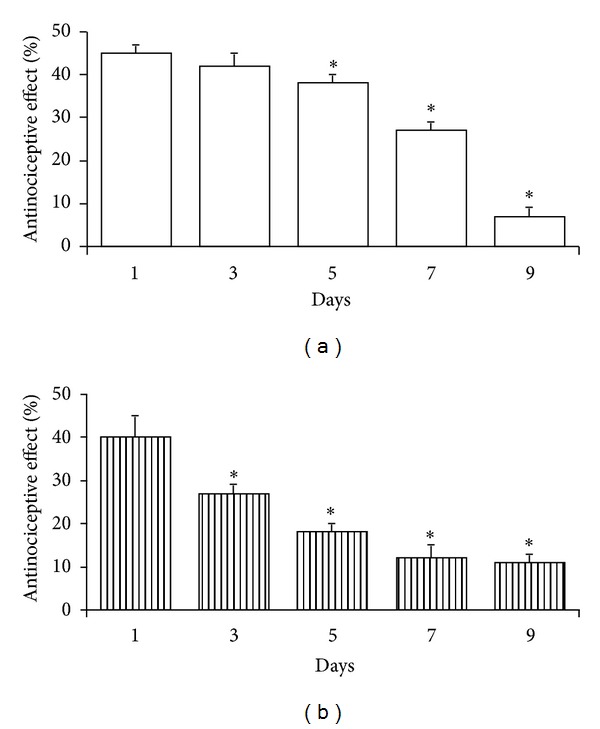
Development of antinociceptive tolerance to chronic ACHC-EM2 given at 20 *μ*g/2 *μ*L twice daily for the indicated time periods. Rats were tested in the tail-flick assay 5 min (a, white columns) and 15 min (b, striped columns) after injection. Mean ± S.E.M.; *n* = 7. Significance was determined by ANOVA; * labels *P* < 0.05 compared to the first day.

In the next set of experiments, rats were repeatedly injected twice daily with ACHC-EM2 (20 *μ*g, *icv*) and the antinociceptive effect was measured 5 and 15 min following injection on various treatment days. It can be seen that the antinociceptive effect gradually decreased with time compared to day 1, which became statistically significant by day 5 and ceased by day 10 showing the development of analgesic tolerance (Figure 2).

### 3.3. Potency and Efficacy of ACHC-EM2 in Activating G-Proteins *In Vitro*

**Figure 3:**
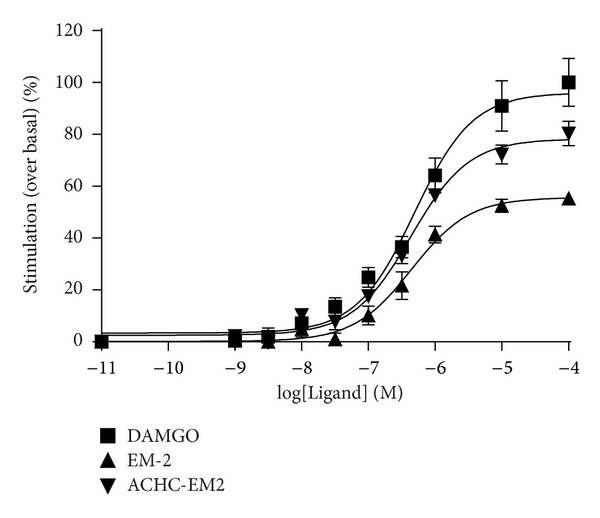
Stimulation of [^35^S]GTP*γ*S binding by opioid peptides in crude rat brain membranes. Membrane homogenates (*≈*10 *μ*g of protein) were incubated with increasing concentrations (10^−9^–10^−4 ^M) of the indicated ligands and [^35^S]GTP*γ*S (0.05 nM) as described in Methods except that 30 *μ*M GDP was used. The curves were fit and drawn by Graph Pad Prism 4 computer program. Results are shown as % stimulation of [^35^S]GTP*γ*S binding over basal values (i.e., binding in the absence of opioid peptides). Each value represents the mean ± S.E.M. of at least three independent experiments performed in triplicate. Nonvisible S.E.M. is within the symbol.

ACHC-EM2 was measured in the ligand-stimulated [^35^S]GTP*γ*S functional assay in crude rat brain membranes and compared to that of EM2 and the prototypic full agonist, DAMGO. Full dose-response curves of the three peptides were assessed between 10^−9^−10^−4 ^M and efficacy and potency values were determined (Figure 3). The *E*_max⁡_ value of DAMGO was set as 100% by definition. The efficacy was significantly lower for both ACHC-EM2 and EM2 with *E*_max⁡_ values of 71.7 ± 1.1 and 52.5 ± 0.8%, respectively. These data show that both ACHC-EM2 and EM2 behave as partial agonists in this assay. The potency of the three peptides in stimulating G-protein activation was very similar, with log⁡⁡EC_50_ value of ACHC-EM2 being 6.37 ± 0.03.

### 3.4. Changes in G-Protein Stimulation due to Chronic Treatments with Mu-Opioid Peptide Agonists

Brains exposed to repeated agonists treatments leading to analgesic tolerance or vehicle were simultaneously processed in every experiment. Control and treated brain homogenates were subjected to subcellular fractionation to obtain highly purified SPM and MI membrane fractions. Functional coupling of mu-opioid receptors to G-proteins was examined by measuring the ability of DAMGO, added to the membrane fractions *in vitro, *to facilitate [^35^S]GTP*γ*S binding (Table 1). The potencies of DAMGO were 44 ± 6 nM and 100 ± 18 nM, and efficacies of 95 ± 5 and 85 ± 9% in control (vehicle administered) SPM and MI, respectively. Chronic treatment with ACHC-EM2 resulted in a significant shift of the dose-response curve of DAMGO to the right; accordingly the EC_50_ values of DAMGO increased to 80 ± 8 nM and 179 ± 25 nM in treated SPM and MI, respectively. Chronic treatment with DAMCK also resulted in attenuated G-protein coupling in the MI fraction. There was a similar tendency in the SPM which, however, did not reach a statistically significant level. Chronic DAMGO treatment did not cause any change in [^35^S]GTP*γ*S incorporation. The efficacies, reflected in the *E*_max⁡_ values, did not change due to any treatments (Table 1).

### 3.5. Changes of Receptor Densities due to Chronic Treatments with Mu-Opioid Peptide Agonists

**Figure 4:**
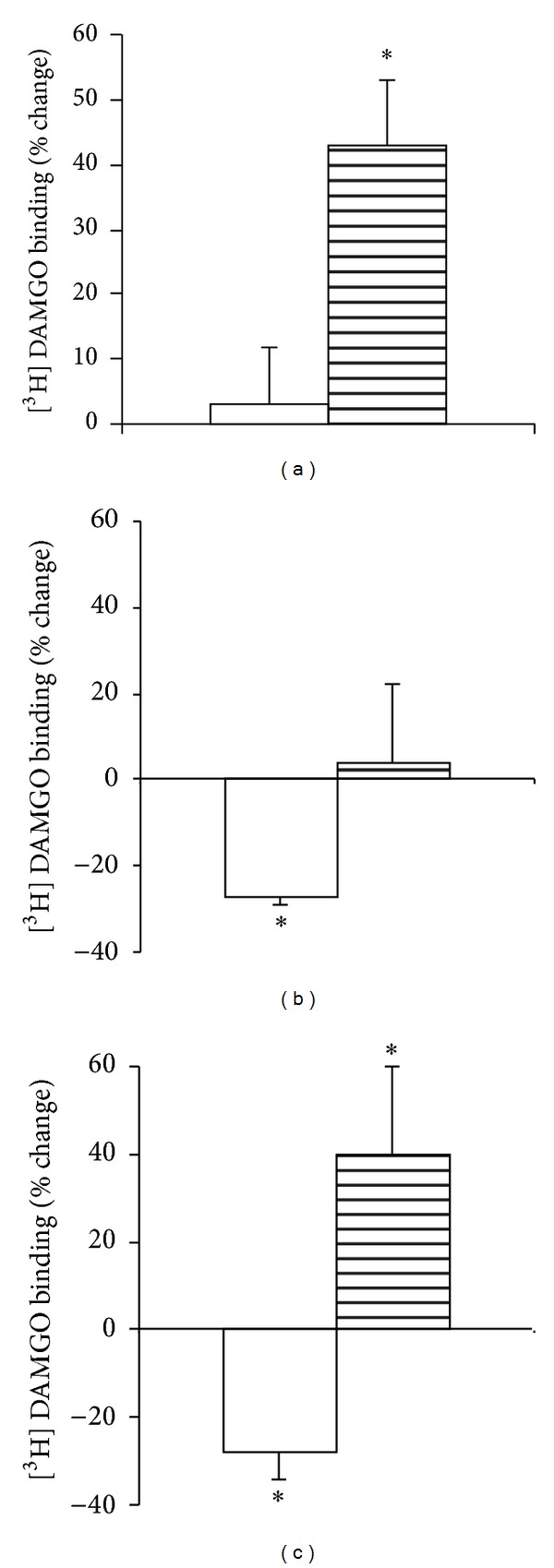
Changes in receptor density (*B*_max⁡_) following chronic exposure of ACHC-EM2 (a) DAMGO (b), and DAMCK (c). SPM (white columns) and MI (striped columns) fractions were prepared from whole brain. The membrane suspensions (0.3 mg protein) were incubated with 1 nM [^3^H]DAMGO for 60 min at 25°C in the absence (total binding) or in the presence of 10^−10^–10^−5 ^M of unlabeled DAMGO. Results are expressed as % change of protein in each fraction. Mean ± S.E.M.; *n* = 3–6; significance was determined by *t*-test; *P < 0.05 compared to control.

 Changes in the binding parameters of mu-opioid receptors were measured in [^3^H]DAMGO homologous displacement experiments (Table 2). As expected, the *K*_*D*_ values were not significantly changed due to acute or chronic ACHC-EM2 treatments. Acute injection of a single dose (20 *μ*g) of the endomorphin derivative did not change the *B*_max⁡_ values in any fractions. Notably, chronic treatment with this peptide resulted in a significant (42%) increase in the density of mu-sites in the MI fraction (Table 2, Figure 4(a)). On the contrary, chronic treatments with the full agonist DAMGO resulted in a 22% decrease in the density of surface mu-sites (Figure 4(b)). Similar effect was detected with the chloromethyl ketone derivative of DAMGO, DAMCK, with a concomitant 40% increase in the MI fractions (Figure 4(c)).

## 4. Discussion

In one part of the work, we have described the pharmacological features of the new endomorphin derivative, ACHC-EM2, upon its *icv* administration in the rat tail-flick test. Acutely, it is slightly more potent than the parent peptide, EM2, having 60 and 45% maximal antinociceptive effect values, respectively (Figure 1). These results are consistent with several investigations in acute pain tests showing that endomorphins exhibited steady plateau at 40% of maximal antinociceptive effect [2, 36–38]. We have shown previously that the prototypic mu-agonist peptide, DAMGO, and its chloromethyl ketone derivative, DAMCK, display profound antinociception of about 80% with distinct time-course. The effect of DAMGO was longer-lasting, which peaked by about 30 min and followed by a rapid dissipation, whereas DAMCK showed an apparently irreversible antinociception [32]. This is in accordance with its irreversible binding to mu-sites in *in vitro *binding assays. It is to be noted that we have *intracerebroventricularly* administrated EM2 and ACHC-EM2, while the antinociceptive effects were examined by using the tail-flick test, mostly a spinal reflex. Opioids are involved in both ascending and descending components of pain modulation. Given supraspinally, multiple descending pain control pathways are involved in antinociception induced by opioid agonists. Interestingly, distinct mechanisms mediating descending pain controls for antinociception induced by supraspinally administered EM1 and EM2 were described in the mouse [39]. 

It has been revealed that full analgesic tolerance develops to the antinociceptive effect of 20 *μ*g ACHC-EM2 by a 10-day treatment (*icv,* twice daily) (Figure 2). For a comparison, tolerance development was also tested for DAMGO and DAMCK. It was found that behavioral tolerance was complete by an 8-day *icv* injection of 10 *μ*g of the latter two peptides (twice daily (data not shown)). For further biochemical studies, the doses of the peptides were designed to maximize tolerance and were not equieffective as performed with other ligands [19].

Since the ligand-stimulated [^35^S]GTP*γ*S functional assay measures receptor mediated G-protein activation, it may be a viable tool to detect adaptations associated with tolerance at the mu-opioid receptor. We have revealed that both EM2 and its new derivative are partial agonists, contrary to the full agonist DAMGO, in the ligand-stimulated [^35^S]GTP*γ*S functional test in crude rat brain membranes (Figure 3). This is in an excellent agreement with our data in the tail-flick analgesia test and recent work, which showed that EM2 has a much lower operational efficacy for G-protein mediated responses than DAMGO at native mu-opioid receptors in mature neurons [40]. However, tolerance to [^35^S]GTP*γ*S incorporation following chronic opioid peptide treatments was not detected in all cases, even when full analgesic tolerance was manifested. It was found that chronic ACHC-EM2 treatment decreased the EC_50_ of DAMGO by about 2-fold showing attenuated coupling of mu-sites to their cognate G-proteins in both SPM and MI membranes. Similar changes were observed upon prolonged DAMCK treatment. On the contrary, chronic DAMGO injection resulted in no significant changes in either the potency or in the efficacy of G-protein signaling (Table 1). The question arises whether a different picture would have been obtained on ACHC-EM2- stimulated [^35^S]GTP*γ*S signaling. Differences in the pattern of G-protein activation have been reported for agonists at mu-opioid receptors, including EM-1 versus EM2 and EM-2 versus DAMGO and morphine [41–43]. In the present work, we did not attempt to identify the G-protein subtypes involved in the effect of each ligand. For detecting desensitization, G-protein activation by the full agonist DAMGO may be a better choice than the partial agonist ACHC-EM2, which may induce either incomplete stimulation of the entire mu-opioid receptor population or full stimulation of only a portion of the entire receptor population. Another reason for testing DAMGO-stimulated [^35^S]GTP*γ*S binding is that the present study is part of a comprehensive work using various mu-opioid ligands of distinct efficacy, chemical nature, and abuse potential to assess regulatory changes due to analgesic tolerance, and this set-up was used in our previous studies [28, 29].

Ligand-specific changes were also detected for receptor trafficking (Figure 4). While chronic DAMGO and DAMCK decreased the density of surface mu-receptors, such changes were not evident by ACHC-EM2. Instead, the density of mu-sites increased by about 40% in the light membrane fraction of ACHC-EM2 tolerant brain homogenates compared to that of vehicle-treated MI fraction (Figure 4(c), Table 2). Previous works from other laboratories have revealed region-specific receptor adaptation in the brain [42, 43]. It is to be considered whether compounds injected via the *icv *route are able to reach the entire population of mu-opioid receptors in the central nervous system. There are reports suggesting that the compounds may remain in the vicinity of the ventricles. On the contrary, *icv *administration of the opioid antagonist beta-funaltrexamine was shown to reduce the density of mu-opioid receptors as measured by *in situ* autoradiography by 40–50% throughout the brain, with little regional variations [44]. Since it is not possible to gain sufficient amount of membrane proteins in subcellular fractions of small tissues, we have measured mu-opioid receptor density in crude membranes. [^3^H]DAMGO binding was downregulated by about 22% in the hippocampus, upregulated by 33% in the spinal cord, and did not significantly change in the cortex and brainstem due to chronic ACHC-EM2 treatments (data not shown).

Overall, distinct regulatory changes were noted for the three opioid peptides, albeit all caused full analgesic tolerance. This is in accordance with a growing number of data showing that different opioid ligands can lead to varying degrees of receptor regulation. The recently discovered phenomenon of “biased agonism” can provide the molecular basis for the observed ligand-specific effects. It is anticipated that different agonists stabilize distinct active conformations of the receptor, thereby inducing distinct downstream signaling [25, 26, 45]. A very recent paper has shown that endomorphins seem to be biased toward arrestin recruitment over G-protein activation contrary to DAMGO [40]. Morphine has been shown to be biased toward *β*-arrestin2 regulation of the mu-opioid receptors. It was published that knocking-out this protein enhanced and prolonged morphine antinociception in the hot-plate test and attenuated tolerance [46]. Although the precise role of arrestins in the regulation of mu-opioid receptors in neurons remains to be fully elucidated, it may explain the observed ligand-specific effects in the present work and others. Functional selectivity may open up new directions for designing potent analgesics with less unwanted side effects.

## 5. Conclusions

Chronic *icv* treatment with the partial agonist endomorphin analog, ACHC-EM2, resulted in the development of analgesic tolerance in the rat tail-flick test. This was accompanied by desensitization of the mu-receptors in subcellular fractions of rat brain. Also, the mu-receptors were upregulated by about 40% in the light membrane fraction with no detectable change in surface binding. The prototypic full mu-agonist peptide, DAMGO, and its chloromethyl ketone derivative, DAMCK, also induced tolerance, yet the accompanying molecular changes were different. These results are in accordance with the recently discovered phenomenon, that is, the so-called “biased agonism” or “functional selectivity”. The new concept may help to identify new lead molecules, which can substitute morphine in pain treatment with improved pharmacological profile.

## Figures and Tables

**Table 1:** Changes in DAMGO-stimulated [^35^S]GTP*γ*S binding induced by *in vivo* chronic opioid peptide treatments in rat brain subcellular fractions.

Treatment	*E*_max⁡_ (% over basal)				EC_50_ (nM)			
	SPM		MI		SPM		MI	
	Control	Treated	Control	Treated	Control	Treated	Control	Treated
ACHC-EM2	95 ± 5	106 ± 5	85 ± 9	109 ± 2	44 ± 6	80 ± 8*	100 ± 18	179 ± 25*
DAMGO	112 ± 12	130 ± 14	60 ± 3	72 ± 9	87 ± 9	107 ± 14	101 ± 14	86 ± 2
DAMCK	112 ± 12	100 ± 9	60 ± 3	61 ± 17	87 ± 9	126 ± 22	101 ± 14	200 ± 42*

ACHC-EM2, DAMGO, and DAMCK were chronically administered to rats as described in Methods. Control animals received CSF. Subcellular fractionation of brain homogenates to obtain synaptic plasma membrane (SPM) and microsomal (MI) fractions was performed. Full concentration curves of DAMGO, consisting of 5-6 concentrations between 10^−8^–10^−4^ M, were measured in [^35^S]GTP*γ*S binding assay. The parameters shown were obtained from nonlinear regression analysis using Graph Pad Prism 4 considering a sigmoidal dose response curve for DAMGO. Results shown are as % stimulation of [^35^S]GTP*γ*S binding over basal values (i.e., binding in the absence of DAMGO). Data are mean ± S.E.M. of 3–6 independent experiments each performed in triplicate. Significant difference between the appropriate values in control and treated membrane fractions was determined by the Student's *t*-test and set as *P < 0.05.

**Table 2:** Changes in [^3^H]DAMGO binding induced by ACHC-EM2 treatments in rat brain subcellular fractions.

Treatment	*K*_D_ (nM)				*B*_max⁡_ (fmol/mg protein)			
	SPM		MI		SPM		MI	
	Control	Treated	Control	Treated	Control	Treated	Control	Treated
ACHC-EM2 acute	5.0 ± 1.9	3.0 ± 1.1	2.3 ± 0.4	3.6 ± 0.9	298 ± 72	258 ± 45	410 ± 59	311 ± 74
ACHC-EM2 chronic	2.9 ± 1.2	1.3 ± 0.2	4.5 ± 2.4	2.5 ± 0.2	291 ± 82	286 ± 50	333 ± 78	462 ± 54*

ACHC-EM2 was injected either acutely or chronically as described in Methods. Control animals received CSF. Subcellular fractionations of brain homogenates to obtain synaptic plasma membrane (SPM) and microsomal (MI) membranes followed by [^3^H]DAMGO binding was performed as outlined in Methods. Data represent the mean ± S.E.M. of at least three independent experiments performed in duplicate. Statistically significant differences due to either treatments compared to appropriate control values in each fraction were determined by Student *t*-test and set at *P < 0.05.
